# Descending serotonergic modulation from rostral ventromedial medulla to spinal trigeminal nucleus is involved in experimental occlusal interference-induced chronic orofacial hyperalgesia

**DOI:** 10.1186/s10194-023-01584-3

**Published:** 2023-05-10

**Authors:** Si-Yi Mo, Yang Xue, Yuan Li, Yao-Jun Zhang, Xiao-Xiang Xu, Kai-Yuan Fu, Barry J. Sessle, Qiu-Fei Xie, Ye Cao

**Affiliations:** 1 Department of Prosthodontics, Center for Oral and Jaw Functional Diagnosis, Treatment and Research, School and Hospital of Stomatology, Peking University, No.22, Zhongguancun South Avenue, Haidian District, Beijing, 100081 PR China; 2 National Center of Stomatology & National Clinical Research Center for Oral Diseases & National Engineering Laboratory for Digital and Material Technology of Stomatology & Beijing Key Laboratory of Digital Stomatology & Research Center of Engineering and Technology for Computerized Dentistry Ministry of Health & NMPA Key Laboratory for Dental Materials, Beijing, 100081 PR China; 3 Center for Temporomandibular Disorders and Orofacial Pain, School and Hospital of Stomatology, Peking University, Beijing, 100081 PR China; 4 Faculty of Dentistry & Department of Physiology, Temerty Faculty of Medicine & Centre for the Study of Pain, University of Toronto, Toronto, ON M5G 1G6 Canada; 5 Key Laboratory for Neuroscience, Ministry of Education/National Health Commission of the People’s Republic of China, Peking University, Beijing, 100083 PR China

**Keywords:** Pain modulation, Serotonin, Rostral ventromedial medulla, Chronic primary myofascial orofacial pain, Experimental occlusal interference, Hyperalgesia

## Abstract

**Background:**

Dental treatment associated with unadaptable occlusal alteration can cause chronic primary myofascial orofacial pain. The serotonin (5-HT) pathway from the rostral ventromedial medulla (RVM) exerts descending modulation on nociceptive transmission in the spinal trigeminal nucleus (Sp5) and facilitates chronic pain. The aim of this study was to investigate whether descending 5-HT modulation from the RVM to the Sp5 is involved in the maintenance of primary myofascial orofacial hyperalgesia after persistent experimental occlusal interference (PEOI) or after delayed removal of experimental occlusal interference (REOI).

**Methods:**

Expressions of 5-HT3A and 5-HT3B receptor subtypes in the Sp5 were assessed by immunofluorescence staining and Western blotting. The release and metabolism of 5-HT in the Sp5 were measured by high-performance liquid chromatography. Changes in the pain behavior of these rats were examined after specific pharmacologic antagonism of the 5-HT3 receptor, chemogenetic manipulation of the RVM 5-HT neurons, or selective down-regulation of 5-HT synthesis in the RVM.

**Results:**

Upregulation of the 5-HT3B receptor subtype in the Sp5 was found in REOI and PEOI rats. The concentration of 5-HT in Sp5 increased significantly only in REOI rats. Intrathecal administration of Y-25130 (a selective 5-HT3 receptor antagonist) dose-dependently reversed the hyperalgesia in REOI rats but only transiently reversed the hyperalgesia in PEOI rats. Chemogenetic inhibition of the RVM 5-HT neurons reversed the hyperalgesia in REOI rats; selective down-regulation of 5-HT in advance also prevented the development of hyperalgesia in REOI rats; the above two manipulations did not affect the hyperalgesia in PEOI rats. However, chemogenetic activation of the RVM 5-HT neurons exacerbated the hyperalgesia both in REOI and PEOI rats.

**Conclusions:**

These results provide several lines of evidence that the descending pathway from 5-HT neurons in the RVM to 5-HT3 receptors in the Sp5, plays an important role in facilitating the maintained orofacial hyperalgesia after delayed EOI removal, but has a limited role in that after persistent EOI.

**Graphical Abstract:**

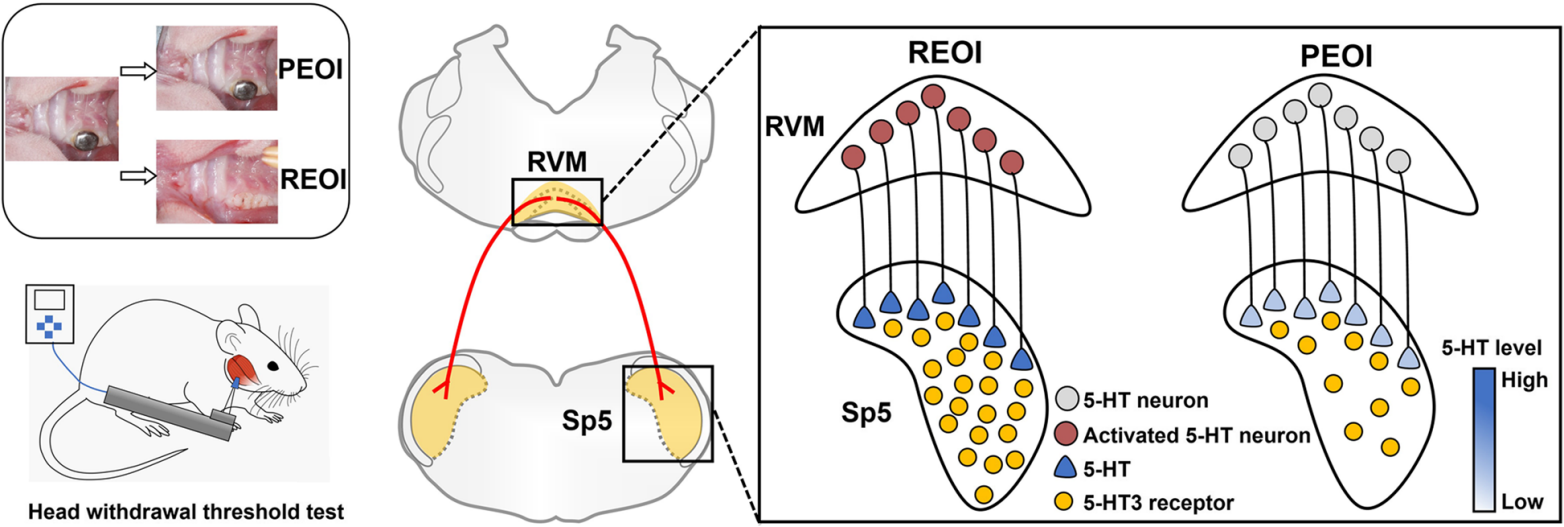

**Supplementary Information:**

The online version contains supplementary material available at 10.1186/s10194-023-01584-3.

## Background

Primary myofascial orofacial pain is a mild to moderate deep aching or pressing pain in the masticatory muscles that may be episodic or unremitting and is often associated with muscular dysfunction [1]. Chronic primary myofascial orofacial pain is a common and refractory condition in clinical practice with few effective treatment strategies [1, 2]. The development of chronic primary myofascial orofacial pain is associated with peripheral [3–5] and central sensitization in the spinal trigeminal nucleus (Sp5) [6–9]. These pathological changes in the ascending nociceptive pathway induced by peripheral inputs may activate the descending modulatory system which in turn influences the ascending nociceptive transmission.

The rostral ventromedial medulla (RVM) is a brainstem region that can promote or suppress the ascending nociceptive transmission by transmitting ultimate pain-facilitating or pain-inhibiting outputs to the spinal dorsal horn and Sp5 [9–11]. There is now considerable evidence that a serotonin (5-HT) pathway from the RVM exerts bidirectional descending modulation on spinal or trigeminal nociceptive transmission and affects nociceptive behavior [12–14]. Increasing attention has been paid to the pain-facilitating role of the 5-HT pathway from the RVM in various pain states [15, 16]. For example, optogenetic activation of RVM 5-HT neurons was found to have a predominant facilitatory effect on pain [17] whereas chemogenetic inhibition of RVM 5-HT neurons abolishes postsurgical pain [16]. Consistent with this finding is the evidence that depletion of 5-HT in the RVM prevents the development of orofacial pain [18, 19]. Furthermore, the spinal 5-HT3 receptor has been shown to mediate pain facilitation among the seven types of 5-HT receptors (5-HT1-7) [15, 20, 21]. The pronociceptive effect of the 5-HT3A receptor subtype has been well demonstrated [15, 22, 23], while the effect of the 5-HT3B receptor subtype has been little studied.

We have previously found that the experimental occlusal interference (EOI) can induce chronic hyperalgesia in the masticatory muscles and that this hyperalgesia simulates the clinical situation of chronic primary myofascial orofacial pain [24]. Furthermore, the EOI-induced myofascial orofacial hyperalgesia was found to be irreversible after delayed removal of EOI (REOI) [25–28]. Electrophysiological recordings have also shown that the predominant facilitatory modulation in the RVM contributes to the development of chronic myofascial orofacial hyperalgesia after delayed REOI, whereas pain inhibition in the RVM predominates over pain facilitation after persistent EOI (PEOI) [28]. These previous findings suggest that the descending modulation from the RVM may differentially influence the development of chronic myofascial orofacial hyperalgesia with or without persistent EOI-related peripheral stimulus. However, whether the 5-HT pathway from the RVM mediates different descending modulatory influences on the Sp5 in these two situations needs further verification.

The aim of this study was to investigate whether descending 5-HT modulation from the RVM to the Sp5 is involved in the maintenance of myofascial orofacial hyperalgesia after PEOI or after delayed REOI. The expression of 5-HT3A and 5-HT3B receptor subtypes and the release and metabolism of 5-HT in the Sp5 were directly assessed in PEOI and REOI rats. Changes in the pain behavior of these rats were also measured after specific pharmacological blockade of the 5-HT3 receptor, chemogenetic manipulation of RVM 5-HT neurons, or selective down-regulation of 5-HT synthesis in the RVM.

## Materials and methods

### Animals

One hundred and fifty-nine adult male Sprague–Dawley rats with an initial weight of 200–220 g were used in this study. The rats were purchased from Vital River Laboratory Animal Technology Company (Beijing, China) and housed in the animal facility of Peking University School and Hospital of Stomatology. The temperature and humidity of the facility were maintained at a constant level and food and water were provided ad libitum. Only male rats were used to avoid estrogenic effects in accordance with our previous studies [24, 27, 28]. The number of rats used in each experimental series is given below in the Experimental design section.

### Model establishment

As previously described, a metal crown was cemented onto the right maxillary first molar of the rat for the application of EOI [24]. For the establishment of PEOI, the EOI was applied and maintained until postoperative day 14. To establish delayed REOI, the EOI was removed with a sharp probe on postoperative day 8 and the rats were allowed to recover until postoperative day 14. Sham rats underwent the same operations but without actual cementation of a metal crown.

### Head withdrawal threshold measurement

As previously described [24], head withdrawal thresholds (HWTs) were tested by applying mechanical stimulation to the ipsilateral or contralateral masseter muscle using a modified testing probe of an electronic von-Frey anesthesiometer (BIO-EVF3, Bioseb, Vitrolles, France). Prior to EOI placement or viral vector injection surgery, rats were placed on the palm of the experimenter's hand and acclimatized to the test environment, without any restraint equipment or methods, to allow them to retract their head freely (Fig. S1). The force applied to the orofacial region was recorded five times to elicit a head withdrawal response, and the average of the five values was calculated as the HWT for that measurement. The average of the HWTs measured on each day of the following 3 days was calculated as the baseline HWT. HWTs measurements were performed in a blinded fashion by the same experimenter.

### Immunofluorescence staining

Rats were deeply anesthetized with 1% sodium pentobarbital (100 mg/kg, i.p.) and subjected to transcardiac perfusion with 250 mL body-temperature saline followed by 300 mL 4% ice-cold paraformaldehyde (Sigma-Aldrich, Darmstadt, Germany). Brainstem tissue (from 1 mm rostral to 2 mm caudal to the obex) was dissected and postfixed in 4% paraformaldehyde at 4 °C overnight. These tissues were then sequentially dehydrated in 20% and 30% sucrose solutions.

Brainstem Sections (20 micron/section) were prepared using a cryostat. Sections from chemogenetics or RNA interference experiment (see below) were mounted on glass slides after sectioning, while other sections from other experiments were placed in the phosphate-buffered saline (0.1 M PBS) for free-floating immunohistochemical staining. After blocking the sections with 10% normal goat serum, incubation of these sections maintained for 2 days at 4 °C with a mixture of primary antibodies (rabbit anti-5-HT3A receptor subtype, 1:100, RRID: AB_838865, Novus Biologicals, Littleton, CO, USA; rabbit anti-5-HT3B receptor subtype, 1:500, RRID: AB_2122423, Abcam, Cambridge, UK; and mouse anti-neuronal-specific nuclear protein, NeuN, 1:2000, RRID: AB_2298772, Millipore, Darmstadt, Germany) or incubated with a mixture of primary antibodies (rabbit anti-Tph2, 1:1000, RRID:AB_2799385; mouse anti-Flag, 1:500, RRID:AB_10950495; Cell Signaling technology, Beverly, MA, USA) overnight at 4 °C. All these sections were incubated with appropriate secondary antibodies for 90 min at room temperature. Secondary antibodies were Alexa 488 or Alexa 594-conjugated goat anti-rabbit or goat anti-mouse IgG (1:200, Zhongshan Golden Bridge Biotechnology, Beijing, China). PBS instead of primary antibody was used as a control stain in selected sections. The stained sections were observed under a fluorescence microscope (BX51, Olympus, Tokyo, Japan) and photographed using a charge-coupled device camera with appropriate filter (DP71, Olympus, Tokyo, Japan).

Double immunofluorescence was performed to verify whether 5-HT3A or 5-HT3B receptor subtype labels were expressed in the neurons. The area and the mean fluorescence intensity of 5-HT3A or 5-HT3B receptor subtype staining of five sections from each rat were semi-quantified (three rats used per group) using Fiji software (NIH, MD, USA). The number of 5-HT3A or 5-HT3B receptor subtype/NeuN double-positive cells and NeuN-positive cells of five sections from each rat (three rats used per group) was counted manually. The number of Tph2-positive cells of five sections from each rat (see below, five rats used per group) in the RNA interference experiment was also manually quantified. For the above semi-quantification and manual quantification, the analyst did not know the grouping information.

### Western blotting

Rats were deeply anesthetized with 1% sodium pentobarbital (100 mg/kg, i.p.) and were decapitated. Brainstem tissue (from 1 mm rostral to 2 mm caudal to the obex) was removed. The Sp5 was quickly harvested, immediately frozen by immersion in liquid nitrogen, and then stored at − 80 °C. Tissues were homogenized in ice-cold RIPA lysis buffer supplemented with protease inhibitor cocktail (Beyotime Biotechnology, Shanghai, China) using a homogenizer (NewZongKe, D1000, Wuhan, China). The homogenates were centrifuged at 14,000 g for 20 min at 4 °C. After collecting the supernatants containing protein lysates, the protein concentration was then determined using a BCA protein assay kit (Beyotime Biotechnology, Shanghai, China). Thirty μg aliquots were subjected to 10% SDS-PAGE gel, and the separated proteins were transferred to polyvinylidene difluoride filters (Millipore, Billerica, MA, USA) by gel electrophoresis. After blocking with 5% non-fat milk for 2 h, the membranes were incubated overnight at 4 °C with primary antibodies against 5-HT3A receptor (1:300, Novus Biologicals, Littleton, CO, USA) or 5-HT 3B receptor (1:500, Abcam, Cambridge, UK). The primary antibody against anti-GAPDH (1:1000, Proteintech, Chicago, USA) was used as an internal control. After extensive washing with Tris-buffered saline containing 0.1% Tween-20, the membranes received incubation in horseradish peroxidase-conjugated secondary antibody (1:1000, Zhong Shan Golden Bridge, Beijing, China) for 1 h to detect antibody-protein complexes. The membranes were then developed in enhanced chemiluminescence solution (Beijing Cowin Biotech Co., Ltd, Jiangsu, China) for 3 min and exposed with a biomolecular imager (LAS-4000 MINI, GE, USA). The density of immunoreactive bands was quantified and normalized using Fiji software.

### High-performance liquid chromatography (HPLC)

Endogenous levels of 5-HT and its metabolite 5-hydroxy indole acetic acid (5-HIAA) were determined using an HPLC system with an electrochemical detector (Model S5300, S1130, Decade Lite, Sykam, Germany). After decapitation of the animal, brainstem tissue (from 1 mm rostral to 2 mm caudal to the obex) was dissected. The Sp5 was quickly removed and immediately frozen by immersion in liquid nitrogen and then stored at − 80 °C until analysis. Tissues were completely homogenized in 0.1 mmol/L ice-cold perchloric acid (by adding 1 mL per 100 mg tissue) for 30 s in a homogenizer (NewZongKE, D1000, Wuhan, China). The sample was centrifuged twice at 14,000 × g for 10 min at 4 °C. Then 20 μL of each supernatant was injected into the HPLC system, onto a C-18 analytical column (2.1 mm × 100 mm; 3 μm) for analysis. The mobile phase (pH 3.0) contained 100 mM sodium dihydrogen phosphate containing 10% methanol, 26 μM EDTA disodium salt dihydrate, and 0.75 mM 1-octanesulfonic acid sodium salt (Sigma-Aldrich, Darmstadt, Germany). The flow rate was adjusted to 0.2 mL/min. The 5-HT and 5-HIAA were detected by oxidation at a glassy carbon electrode with an applied potential of + 750 mV against an Ag/AgCl reference electrode and quantified by an external standard method. The concentrations of 5-HT and 5-HIAA were calibrated by detecting the standard 5-HT and 5-HIAA at several different concentrations (0, 20, 40, 60, 80, 100 ng/mL) respectively. The turnover rate of 5-HT was calculated by dividing the concentration of 5-HIAA by the concentration of 5-HT. The concentration of 5-HIAA added to the concentration of 5-HT of each sample was calculated as the total concentration of 5-HT and 5-HIAA.

### Intrathecal administration of drugs

Each rat was anesthetized with isoflurane inhalation (5% for induction, 2 ~ 2.5% for maintenance). A 25-gauge, 1-in. disposable needle was connected to a 25 μL Hamilton syringe via a polyethylene catheter. A 10 μL solution containing 4.8, 24, or 48 μg of a selective 5-HT3 receptor antagonist Y-25130 (Tocris Bioscience, Bristol, UK) [29] or artificial cerebrospinal fluid (aCSF) [27] was injected intrathecally (i.t.) into the intervertebral space between L4 and L5 vertebrae within 2 min. Any residual drug was slowly flushed out with 10 μL of aCSF. The Y-25130 was initially dissolved in aCSF at a concentration for storage and then diluted in aCSF just before its use. The doses of i.t. Y-25130 administration were chosen on the basis of results from previous studies [29, 30].

### Surgery for viral injection into the RVM

After general anesthesia with sodium pentobarbital (50 mg/kg, i.p.), the rat head was fixed in a brain stereotaxic apparatus (Model 940, David Kopf Instrument, Tujunga, CA, USA). After the skull was exposed by a midline scalp incision, the target injection site was positioned using the stereotaxic apparatus and a small hole was made in the skull above the target site by using a handheld drill. The stereotaxic coordinate of the injection site was 10.92 mm posterior to the bregma, 0 mm lateral to the midline, and 10.6 mm inferior to the bregma. A glass microelectrode filled with paraffin oil was installed into a glass microelectrode injection pump (R480, Reward, Shenzhen, China). A volume of 500 nL of virus was absorbed into the glass microelectrode. The pump was then started to inject the virus into the RVM zone at a rate of ~ 50 nL/min. The injection volume of all the viruses was 400 ~ 500 nL/site. After a 10-min delay, the glass microelectrode was slowly withdrawn. After injection, the viruses were allowed to express in the RVM for 3–4 weeks.

### Chemogenetics

We used a chemogenetic technique with a designer receptor exclusively activated by designer drugs (DREADDs) strategy to manipulate the activity of 5-HT neurons. Clozapine oxide (CNO, 3 mg/kg, Apexbio, Houston, USA) was injected intraperitoneally (i.p.) as the designer drug to activate the designer receptor. AAV2/9-Tph2-hM4Di-mCherry virus (titer: 2.17 × 10^12^ v.g./mL, BrainVTA, Wuhan, China) or AAV2/9-Tph2-hM3Dq-3xflag virus (titer: 2.78 × 101^2^ v.g./mL, BrainVTA, Wuhan, China) were injected into the rat RVM zone to selectively inhibit or activate the RVM 5-HT neurons. AAV2/9-Tph2-mCherry virus (titer: 2.62 × 10^12^ v.g./mL, BrainVTA, Wuhan, China) was injected into the RVM zone as a control. The mCherry gene in the AAV2/9-Tph2-hM3Dq-3xflag virus was replaced by the 3xFlag gene which encodes a type of tag protein due to the limited viral gene capacity. Mouse anti-Flag antibody was used to mark the expression of AAV2/9-Tph2-hM3Dq-3xflag virus. The viral genome of these viruses contains the tryptophan hydroxylase 2 (Tph2) promoter, allowing these viruses to specifically infect and express in 5-HT neurons.

### RNA interference

The shRNA plasmids for Tph2 were used to design the enclosed shRNA (Tph2: TCAACATGCTCCATATTGAAT) [31]. A scrambled shRNA was designed as a control. These plasmids were packaged into two vector viruses respectively, AAV2/9-hsyn-EGFP-5’miR-30a-shRNA(Tph2)-3’miR virus (titer: 1.03 × 10^12^ v.g./mL, BrainVTA, Wuhan, China) and AAV2/9-hsyn-EGFP-5’miR-30a-shRNA(scramble)-3’miR virus (titer: 5.31 × 10^11^ v.g./mL, BrainVTA, Wuhan, China). The viruses were injected into the RVM region and allowed 3–4 weeks to express.

### Experimental design

A total of 159 rats were used in this study. To assess the release of 5-HT and expression of 5-HT3 receptors in the Sp5, 39 of these rats were assigned to sham, REOI, and PEOI groups randomly. Nine rats (*n* = 3 per group) were employed in the immunofluorescence staining experiment, 15 rats (*n* = 5 per group) were used in the Western blotting experiment, and 15 rats (*n* = 5 for each group) were included in the HPLC experiment.

To verify the role of the 5-HT3 receptor in the maintenance of hyperalgesia, 42 of these rats were divided into sham, REOI, and PEOI groups randomly and received pharmacological antagonism of the 5-HT3 receptor. The sham rats received i.t. 48 μg Y-25130 administration (*n* = 6), the REOI rats received i.t. 48, 24, 4.8 μg Y-25130 administration (*n* = 6 for each dose) or aCSF administration (*n* = 6), while the PEOI rats received i.t. 48 μg Y-25130 administration (*n* = 6) or aCSF administration (*n* = 6). The drug was injected intrathecally for 6 consecutive days (from postoperative day 8 to 13). The HWTs were measured before EOI application (baseline), on postoperative days 13, 14, 16, 18, and 21 after EOI application. Since the hyperalgesia of PEOI rats could not be chronically reversed by the highest dose of i.t. Y-25130 used in preliminary experiments, the middle and lowest doses of i.t. Y-25130 were omitted in PEOI rats and sham rats to save on animal numbers.

To test the function of the RVM 5-HT neurons in the maintenance of hyperalgesia, 48 rats were used in the chemogenetic experiments and randomly divided into REOI and PEOI groups. Both the REOI and PEOI rats received hM4Di, hM3Dq, or mCherry virus injection respectively (see above) prior to model establishment, with 8 rats used for each virus type. HWTs were measured before virus injection (baseline), before EOI application (day 0), before CNO injection on day 14 and at 30 min, 6 h, and 24 h after CNO injection on day 14. The change in HWT (ΔHWT) was calculated by subtracting the HWT before CNO injection from the HWT at 30 min after CNO injection on day 14. After completion of the HWT measurements, all the rats were sacrificed. Expression levels of the relevant viruses, injection sites, and specificity of the Tph2 promoter were verified by immunofluorescence staining. Due to misplaced microinjection or lack of mCherry or 3xFlag expression, data from seven of these 48 rats were excluded.

To evaluate the role of 5-HT synthesized in the RVM in the development of orofacial hyperalgesia, 30 rats were divided into REOI and PEOI groups randomly in the RNA interference experiment. Prior to EOI application, Tph2-shRNA (*n* = 9) or scramble-shRNA (*n* = 7) viruses were injected into the RVM (see above) respectively in both REOI and PEOI rats. HWTs were measured before virus injection (baseline), before EOI application (day 0), and on day 14 and day 21. After the measurement of HWTs, the rats were sacrificed. The RVM tissues were dissected (2–4 mm rostral to the obex) and postfixed in 4% paraformaldehyde. Immunofluorescence staining was performed to confirm the expression of relevant viruses, the accuracy of injection sites, and the expression level of Tph2 protein. Data from one rat were excluded because of misplaced microinjection.

### Statistical analyses

Results are presented as the mean ± SD. SPSS v20.0 (SPSS, Chicago, IL, USA) was used for statistical analysis. One-way ANOVA followed by Tukey’s post hoc test was used to analyze data from the immunofluorescence staining, Western blotting, and HPLC experiments. Two-way or three-way repeated measures ANOVA followed by Tukey’s post hoc test was executed to analyze the HWT data. Cell numbers in the RNA interference experiment were compared using two-way ANOVA followed by Tukey’s post hoc test. The sample sizes were set based on previous studies [7, 32–34] and the G*Power 3.1 software [35]. Three to five rats per group were determined in the molecular detection testing or histological verification experiments, 6 to 10 rats per group was estimated in different HWT testing experiments. All statistical analyses, *P*-value, F-value and sample size/group for each figure are listed in Table S1. The significance level was set at *P* < 0.05.

## Results

### Both delayed EOI removal and persistent EOI upregulated the expression level of the 5-HT3B receptor subtype in the Sp5

**Fig. 1:**
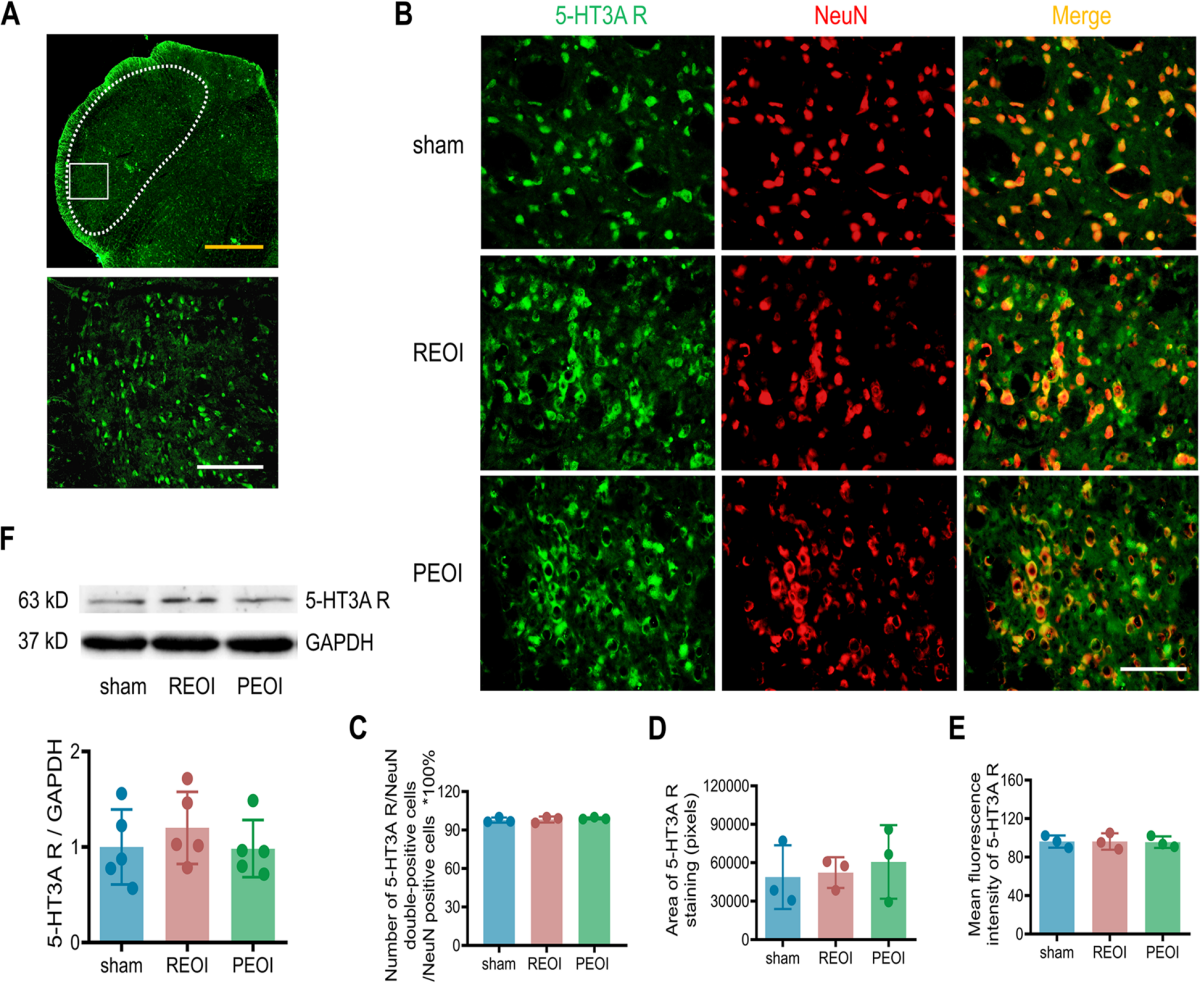
Neither delayed EOI removal nor persistent EOI upregulated the 5-HT3A receptor subtype in the Sp5. **A** Upper panel shows low magnification photograph of 5-HT3A receptor subtype immunostaining in the Sp5 brainstem section of the sham rat. Scale bar, 500 μm. Lower panel shows representative high-magnification photograph of 5-HT3A receptor subtype immunostaining in the area encircled by a white rectangle (ventrolateral region) in the upper panel. Scale bar, 200 μm. **B** Representative high-magnification photographs showing co-expression of 5-HT3A receptor subtype with NeuN in the Sp5 in sham, REOI, and PEOI rats. Scale bar, 50 μm. **C**-**E** Semi-quantitative analysis on the ratio of 5-HT3A receptor subtype/NeuN double-positive cells to NeuN-positive cells, the area of 5-HT3A receptor subtype staining, and mean fluorescence intensity of 5-HT3A receptor subtype showed no significant change in REOI and PEOI rats compared with sham rats (*n* = 3 rats per group, each symbol represents one rat with five sections measured per rat). **F** Western blotting illustrated no change in 5-HT3A receptor subtype expression in either REOI or PEOI rats in the Sp5 (*n* = 5 rats per group). Results represent mean ± SD; one-way ANOVA followed by Tukey’s multiple-comparison test. 5-HT3A R, 5-HT3A receptor subtype

We investigated whether delayed EOI removal or persistent EOI could induce the upregulation of the 5-HT3A and 5-HT3B receptor subtypes in the Sp5. Immunofluorescence labeling and Western blotting for the 5-HT3A and 5-HT3B receptor subtypes were examined in sham, REOI, and PEOI rats on postoperative day 14. The 5-HT3A receptor subtype colocalized with the neurons in the Sp5, including subnucleus caudalis (Sp5C), subnucleus interpolaris/subnucleus caudalis transition zone (Sp5I/Sp5C), and subnucleus interpolaris (Sp5I) (Fig. 1A, B). Over 90% of the 5-HT3A receptor subtype-positive cells colocalized with NeuN-positive cells (Fig. 1C). Semi-quantitative analysis on the ratio of 5-HT3A receptor subtype/NeuN double-positive cells to NeuN-positive cells, the area of 5-HT3A receptor subtype staining, and the mean fluorescence intensity of the 5-HT3A receptor subtype showed no significant change in either REOI or PEOI rats compared to sham rats (Fig. 1C-E). No significant upregulation of the levels of 5-HT3A receptor subtype was found in the Sp5 of REOI and PEOI rats compared to sham rats (Fig. 1F; Fig. S2).

**Fig. 2:**
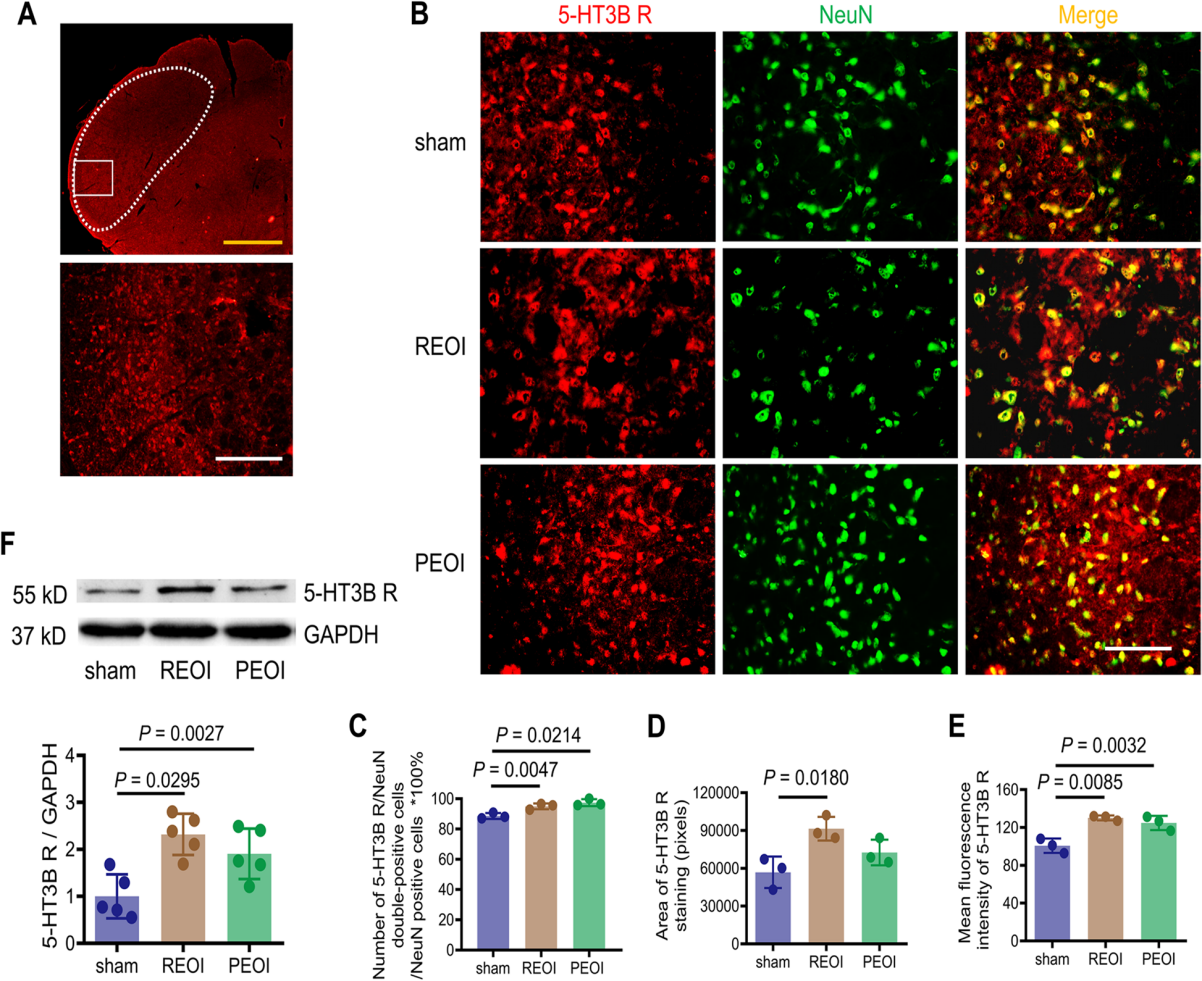
Both delayed REOI and PEOI upregulated the 5-HT3B receptor subtype in the Sp5. **A** Upper panel shows low magnification photograph of 5-HT3B receptor subtype immunostaining in the Sp5 brainstem section of the sham rat. Scale bar, 500 μm. Lower panel shows representative high-magnification photograph of 5-HT3B receptor subtype immunostaining in the area encircled by a white rectangle (ventrolateral region) in the upper panel. Scale bar, 200 μm. **B** Representative high-magnification photographs showing the co-expression of 5-HT3B receptor subtype with NeuN in the Sp5 in sham, REOI, and PEOI rats. Scale bar, 50 μm. **C** Semi-quantitative analysis on the ratio of 5-HT3B receptor subtype/NeuN double-positive cells to NeuN-positive cells showed a significant increase in REOI and PEOI rats (*n* = 3 rats per group, each symbol represents one rat with five measured sections per rat). (D) Semi-quantitative analysis on the area of 5-HT3B receptor subtype showed a significant increase in REOI rats (*n* = 3 rats per group, each symbol represents one rat with five sections measured per rat). **E** Semi-quantitative analysis on the mean fluorescence intensity of 5-HT3B receptor subtype showed a significant increase in REOI and PEOI rats (*n* = 3 rats per group, each symbol represents one rat with five measured sections per rat). **F** Western blotting illustrated increased expression levels of 5-HT3B receptor subtype in REOI and PEOI rats (*n* = 5 rats per group). Results represent mean ± SD; one-way ANOVA, followed by Tukey’s multiple-comparison test. 5-HT3B R, 5-HT3B receptor subtype

The 5-HT3B receptor subtype also colocalized with the neurons in the Sp5C, Sp5I/Sp5C, and Sp5I (Fig. 2A, B). A marked increase in 5-HT3B receptor subtype expression was observed in REOI and PEOI rats (Fig. 2B). Further analysis showed that the ratio of 5-HT3B receptor subtype/NeuN double-positive cells to NeuN-positive cells was higher in REOI (94.97%) and PEOI (97.40%) rats compared to sham rats (88.60%) (Fig. 2C). The area of 5-HT3B receptor subtype staining was significantly increased in REOI rats (Fig. 2D). The mean fluorescence intensity of 5-HT3B receptor subtype was significantly increased in both REOI and PEOI rats (Fig. 2E). In addition, the expression level of 5-HT3B receptor subtype was significantly upregulated in the Sp5 of REOI and PEOI rats (Fig. 2F; Fig. S2). Both the GABAergic (inhibitory interneurons) and glutaminergic neurons (excitatory interneurons) in the Sp5 receiving input from the RVM colocalized with 5-HT3B receptors (Fig. S3).

### Delayed EOI removal, but not persistent EOI, increased the concentration of 5-HT in the Sp5

**Fig. 3:**
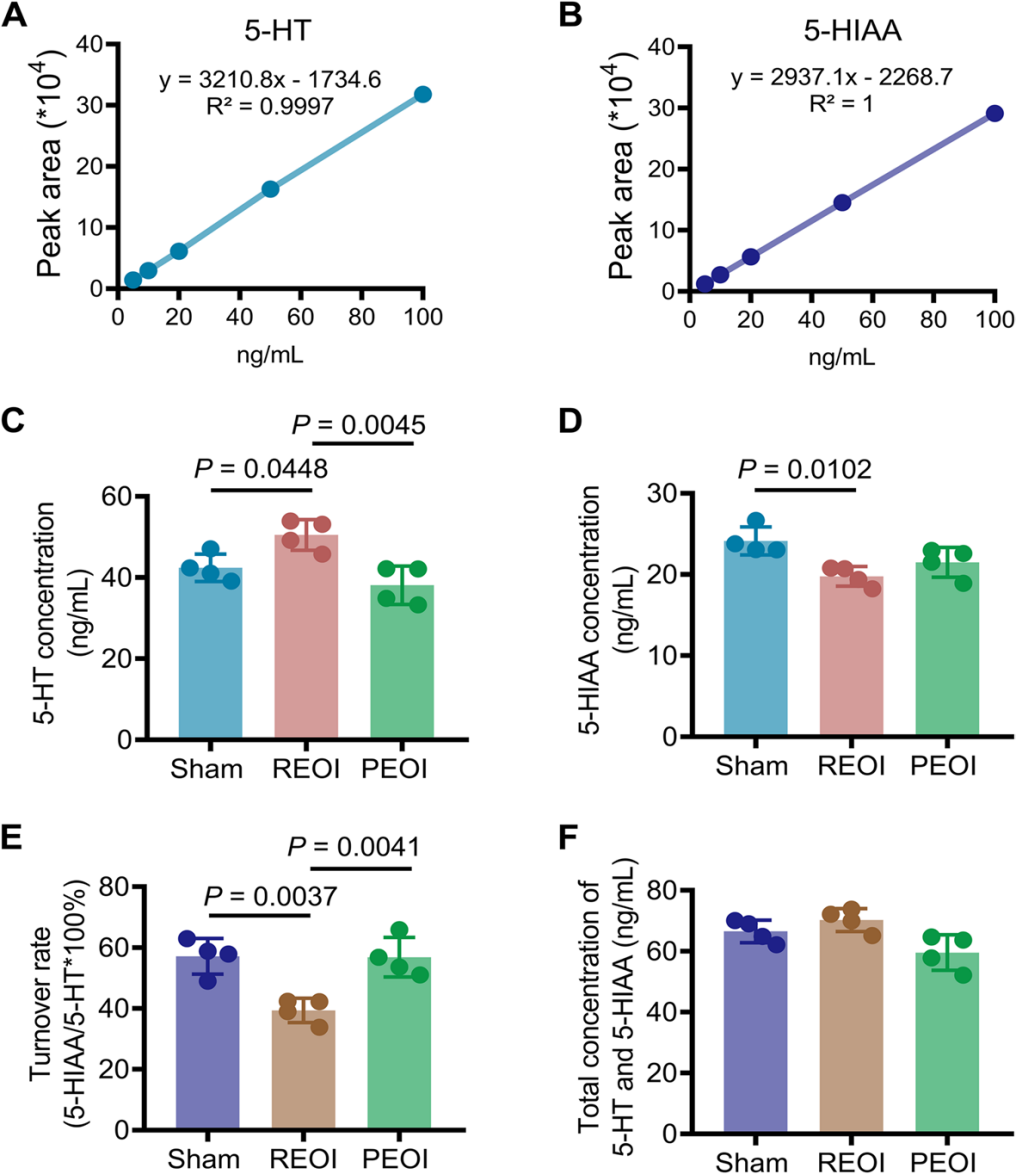
Delayed REOI, but not persistent EOI, increased the concentration of 5-HT in the Sp5. **A**, **B** The calibration curve of 5-HT and 5-HIAA concentration fitted by the detection of standard 5-HT and 5-HIAA, respectively, at various concentrations. **C** The 5-HT concentration was increased in REOI rats. **D** The 5-HIAA concentration was decreased in REOI rats. **E** The turnover rate was reduced in REOI rats. **F** The total concentration of 5-HT and 5-HIAA was not changed in both REOI and PEOI rats. *n* = 4 for each group. Results represent mean ± SD; one-way ANOVA, followed by Tukey’s multiple-comparison test

To assess the impact of delayed EOI removal or persistent EOI on 5-HT release in the Sp5, the concentrations of 5-HT and its metabolite 5-HIAA were measured (Fig. 3A, B). In REOI rats, the concentration of 5-HT increased significantly (Fig. 3C) while the concentration of 5-HIAA decreased significantly (Fig. 3D). In PEOI rats, the concentration of 5-HT and 5-HIAA were not changed (Fig. 3C, D). The turnover rate of 5-HT was significantly decreased in REOI rats but not in PEOI rats (Fig. 3E). However, the total concentrations of 5-HT and 5-HIAA were not significantly different among all groups (Fig. 3F).

### Antagonizing 5-HT3 Receptors Dose-dependently Reversed the Hyperalgesia after delayed EOI removal but only transiently reversed the hyperalgesia associated with persistent EOI

**Fig. 4:**
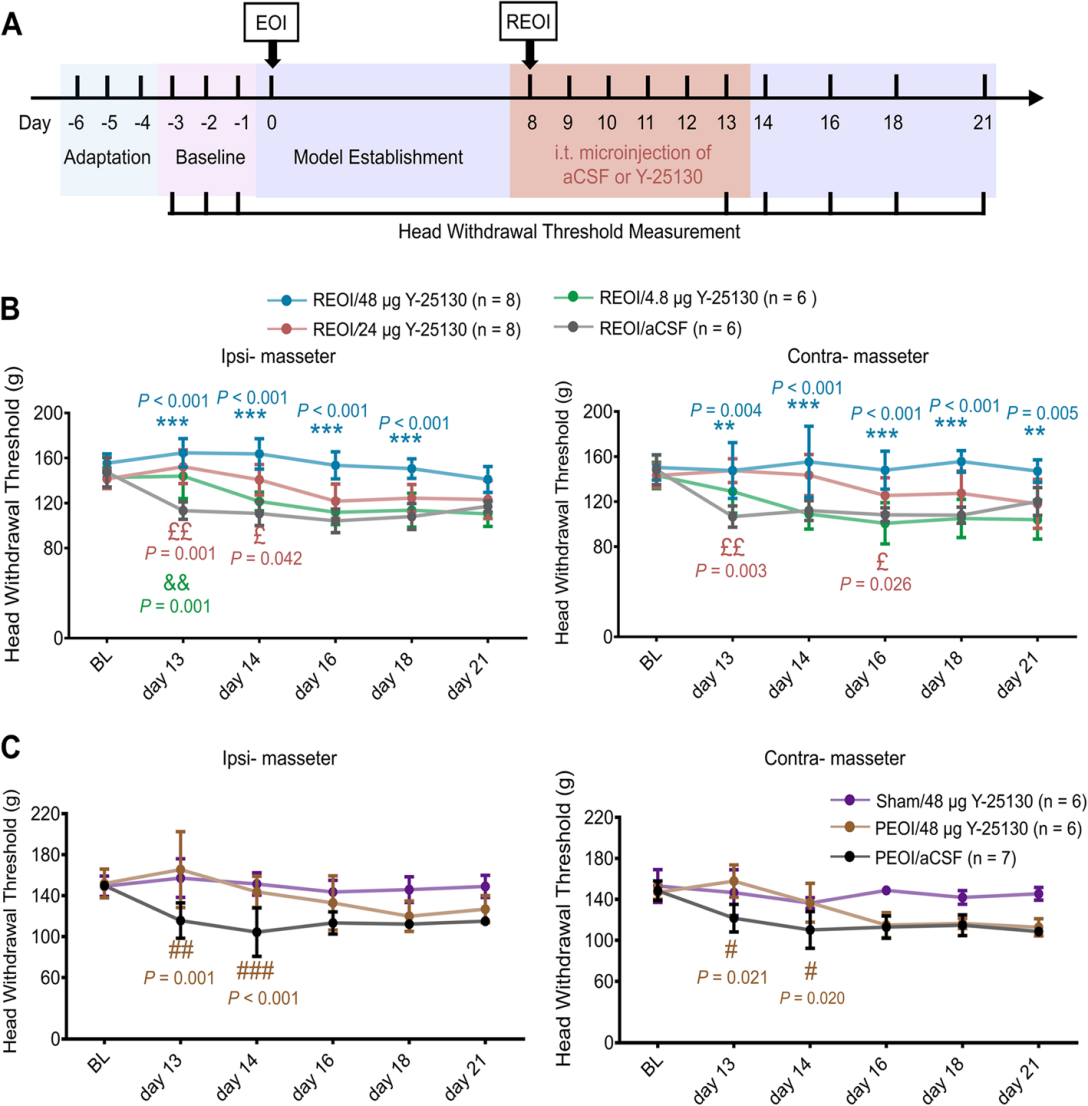
Y-25130 dose-dependently reversed the hyperalgesia after delayed REOI but transiently reversed PEOI-induced hyperalgesia. **A** Schematic diagram of the experimental time course of HWT measurements. The rats received adaption on days 4–6 prior to EOI application. Baseline HWT was obtained by measuring the average HWTs for 3 consecutive days before EOI application. REOI and PEOI models were established from day 0. On days 8–13, rats received consecutive i.t. microinjections of aCSF or 5-HT3 receptor antagonist Y-25130. HWTs related to mechanical stimulation of ipsilateral and contralateral orofacial sides (*n* = 6 per group) were measured at different time points. **B** Changes in ipsilateral and contralateral HWTs in REOI rats injected with 48, 24, 4.8 μg Y-25130, or aCSF. Injection of 48, 24, and 4.8 μg Y-25130 dose-dependently reversed the reduced HWTs in REOI rats. Results represent mean ± SD; three-way repeated measures ANOVA, followed by Tukey’s multiple-comparison test. *P* < 0.001, for days 13, 14, 16, 18, and 21 vs. baseline in the REOI/aCSF group. ** *P* < 0.01, *** *P* < 0.001, the REOI/48 μg Y-25130 group vs. the REOI/aCSF group. £ *P* < 0.05, ££ *P* < 0.01, the REOI/24 μg Y-25130 group vs. the REOI/aCSF group. && *P* < 0.01, the REOI/4.8 μg Y-25130 group vs. the REOI/aCSF group. **C** Changes in ipsilateral and contralateral HWTs in PEOI rats injected with 48 μg Y-25130 or aCSF and sham rats injected with 48 μg Y-25130. Injection of 48 μg Y-25130 only transiently reversed the reduced HWTs in PEOI rats. Results represent mean ± SD; three-way repeated measures ANOVA, followed by Tukey’s multiple-comparison test. *P* < 0.001, for days 13, 14, 16, 18, and 21 vs. baseline in the PEOI/aCSF group. # *P *< 0.05, ## *P* < 0.01, ### *P* < 0.001, the PEOI/48 μg Y-25130 group vs. the PEOI/aCSF group. BL, baseline. HWT, head withdrawal threshold. Tph2, tryptophan hydroxylase 2

To investigate the involvement of 5-HT3 receptors in the Sp5 in the orofacial hyperalgesia after delayed EOI removal or persistent EOI, the effect of i.t. administration of 5-HT3 receptor antagonist on the hyperalgesia was tested by behavioral test (Fig. 4A). Behavioral tests were in the sham (receiving 48 μg Y-25130), REOI (receiving 48 μg, 24 μg, 4.8 μg Y-25130 or aCSF) and PEOI (receiving 48 μg Y-25130 or aCSF) groups.

For REOI rats, the factors of time (*F*_*(5, 36)*_ = 4.289, *P* = 0.004) and drug (*F*_*(3, 40)*_ = 4.340, *P* = 0.010) as well as their interaction (*F*_*(15, 114)*_ = 1.457, *P* < 0.001) showed significant main effects on the HWTs, but the main effect of testing side (*F*_*(1, 40)*_ = 1.660, *P* = 0.205) was not significant. Thus, the HWTs with respect to two testing sides (ipsilateral or contralateral) showed no significant difference (Fig. 4B). The HWTs were significantly reduced and this reduction persisted until day 21 in the REOI/aCSF group (Fig. 4B). The reduced HWTs were significantly reversed after consecutive i.t. 48 μg Y-25130 administrations in REOI/48 μg Y-25130 group compared to the HWTs in the REOI/aCSF group and this reversal could last until day 18 ipsilaterally and day 21 contralaterally (Fig. 4B). However, compared with the HWTs in the REOI/aCSF group, i.t. administration of 24 μg Y-25130 transiently reversed the reduced HWTs in the REOI/24 μg Y-25130 group and this reversal lasted until day 14 ipsilaterally and day 16 contralaterally, whereas i.t. administration of 4.8 μg Y-25130 could only transiently reverse the reduced HWTs in the REOI/4.8 μg Y-25130 group on day 13 ipsilaterally (Fig. 4B).

For PEOI and sham rats, the main effects of the factors of time (*F*_*(4.503, 28)*_ = 28.754, *P* < 0.001) and drug (*F*_*(2, 32)*_ = 25.308, *P* < 0.001), as well as their interaction (*F*_*(9.006, 58)*_ = 10.580, *P* < 0.001) on the HWTs were significant, while the main effect of testing side (*F*_*(1, 32)*_ = 1.636, *P* = 0.210) was not significant. Thus, the HWTs with respect to two testing sides (ipsilateral or contralateral) showed no significant difference either in PEOI or sham rats (Fig. 4C). The HWTs were also significantly reduced and this reduction persisted until day 21 in the PEOI/aCSF group (Fig. 4C). The reduced HWTs were only transiently reversed in the PEOI/48 μg Y-25130 group after consecutive i.t. 48 μg Y-25130 administration compared with the HWTs in the PEOI/aCSF group; the reversal lasted only until day 14 both ipsilaterally and contralaterally (Fig. 4C). Administration of 48 μg Y-25130 did not change the ipsilateral or contralateral HWTs in sham rats either (Fig. 4C).

### Manipulating 5-HT neurons regulated hyperalgesia after delayed EOI removal and hyperalgesia associated with persistent EOI

**Fig. 5:**
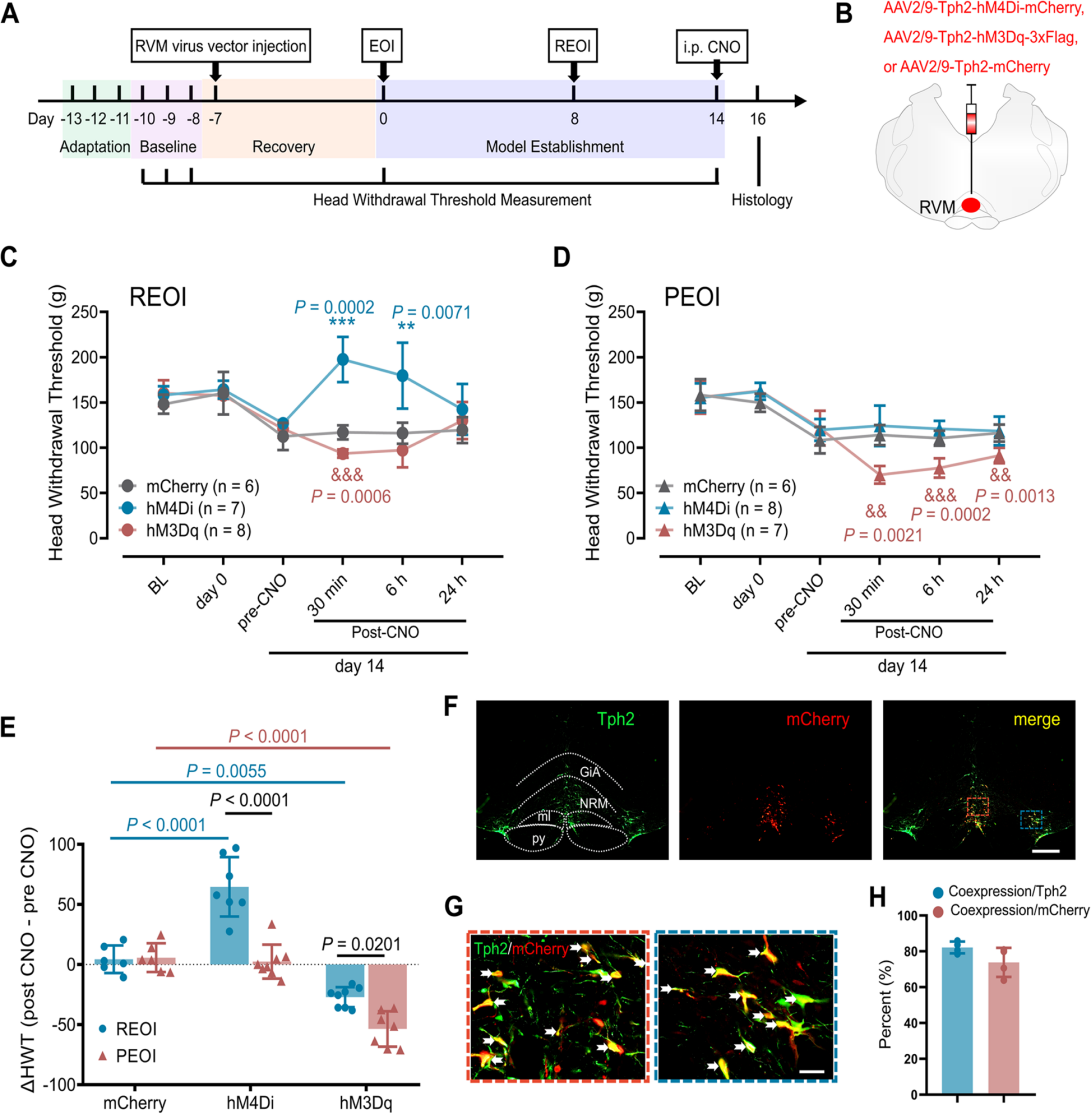
Chemogenetic manipulation of RVM 5-HT neurons regulated the hyperalgesia after delayed REOI and PEOI-induced hyperalgesia. **A** Schematic diagram of the experimental time course of HWT measurements (*n* = 6–8 in each group). **B** Injection of viruses into the RVM region. **C** Changes in HWTs in REOI rats expressing hM4Di, hM3Dq, or mCherry respectively. Results represent mean ± SD; two-way repeated measures ANOVA, followed by Tukey’s multiple-comparison test. *P* = 0.0013, 0.0057, and 0.0001 for the mCherry-expressing, hM4Di-expressing, or hM3Dq-expressing REOI groups, respectively, before CNO injection on day 14 vs. baseline. ** *P* < 0.01, *** *P* < 0.001, the hM4Di-expressing REOI group vs. the mCherry-expressing REOI group. &&& *P* < 0.001, the hM3Dq-expressing REOI group vs. the mCherry-expressing REOI group. (D) Changes in HWTs in PEOI rats expressing hM4Di, hM3Dq, or mCherry respectively. Results represent mean ± SD; two-way repeated measures ANOVA, followed by Tukey’s multiple-comparison test. *P* = 0.0055, 0.0122, and 0.0339 for the mCherry-expressing, hM4Di-expressing, or hM3Dq-expressing PEOI groups, respectively, before CNO injection on day 14 vs. baseline. && *P* < 0.01, &&& *P* < 0.001, the hM3Dq-expressing PEOI group vs. the mCherry-expressing PEOI group. **E** ΔHWT in REOI or PEOI rats expressing hM4Di, hM3Dq or mCherry respectively. Results represent mean ± SD; two-way ANOVA, followed by Tukey’s multiple-comparison test. **F** Representative images of mCherry expression in the RVM and the histological verification of the injection site. Scale bar, 500 μm. GiA, nucleus gigantocellularis pars α; NRM, nucleus raphe magnus; ml, medial lemniscus; Py, pyramidal tract. **G** High magnification photographs of the area encircled in red or blue rectangle respectively in **F** show the co-expression of Tph2 with mCherry in the RVM (the white arrows). Scale bar, 50 μm. **H** The infection rate and specificity of the viruses in the RVM were examined by histological verification. CNO, clozapine. Tph2, tryptophan hydroxylase 2

To assess the influences of RVM 5-HT neurons in the regulation of hyperalgesia after delayed EOI removal or after persistent EOI, we further chemogenetically manipulated the activities of RVM 5-HT neurons to test their effects on the hyperalgesia. Behavioral tests were conducted accordingly in REOI and PEOI groups before and after injection of three different chemogenetic viruses (hM4Di, hM3Dq, and mCherry) into the RVM (Fig. 5A, B).

In REOI rats, the HWTs were not changed after virus injection surgery but had decreased significantly before CNO injection on day 14 (Fig. 5C). The main effects of the factors of time (*F*_*(3.43, 61.74)*_ = 21.85, *P* < 0.0001), virus type (*F*_*(2, 18)*_ = 26.97, *P* < 0.0001), and their interaction (*F*_*(10, 90)*_ = 17.25, *P* < 0.0001) on the HWTs of REOI rats were significant. In the mCherry-expressing REOI group, the reduced HWT before CNO injection on day 14 was not significantly changed after CNO injection (Fig. 5C). However, the reduced HWT before CNO injection in the hM4Di-expressing REOI group was significantly reversed in comparison with the HWT of the mCherry-expressing REOI group at 30 min and 6 h post-CNO injection on day 14; this reversal was not significant at 24 h post-CNO injection (Fig. 5C). The reduced HWT before CNO injection on day 14 of the hM3Dq-expressing REOI group was significantly further reduced compared to the HWT of the mCherry-expressing REOI group only at 30 min post-CNO injection but was not changed at 6 h and 24 h post-CNO injection (Fig. 5C).

In PEOI rats, the HWTs were not changed after the virus injection surgery but had decreased significantly before CNO injection on day 14 (Fig. 5D). The factors of time (*F*_*(3.757, 67.63)*_ = 93.72, *P* < 0.0001) and virus type (*F*_*(2, 18)*_ = 13.71,* P* < 0.0002) showed significant main effects on the HWTs of PEOI rats, as well as their interaction (*F*_*(10, 90)*_ = 9.708,* P* < 0.0001). In both the mCherry-expressing and hM4Di-expressing PEOI groups, the reduced HWTs were not changed after CNO injection compared with the HWTs before CNO injection on day 14 (Fig. 5D). However, the reduced HWT of the hM3Dq-expressing PEOI group on day 14 was significantly further decreased in comparison with the HWT of the mCherry-expressing PEOI group at 30 min, 6 h, and 24 h post-CNO injection (Fig. 5D).

Furthermore, the ΔHWT (HWT at 30 min post-CNO – HWT before-CNO) at day 14 approached 0 in the mCherry-expressing REOI group, the mCherry-expressing PEOI group, and the hM4Di-expressing PEOI group, suggesting that CNO injection did not alter the HWTs at day 14 in these rats (Fig. 5E). The main effects of the factors of EOI treatment (*F*_*(1, 36)*_ = 38.46, *P* < 0.0001) and virus type (*F*_*(2, 36)*_ = 90.51, *P* < 0.0001) on the ΔHWT were significant, as was their interaction (*F*_*(2, 36)*_ = 14.97, *P* < 0.0001). Interestingly, the ΔHWT was significantly increased in hM4Di-expressing REOI group (vs. the mCherry-expressing REOI group), suggesting that CNO injection may have led to a reversal of the reduced HWT on day 14 (Fig. 5E). However, the ΔHWT was significantly decreased in both the hM3Dq-expressing REOI group (vs. the mCherry-expressing REOI group) and the hM3Dq-expressing PEOI group (vs. the mCherry-expressing PEOI group), indicating that a further decrease in HWT occurred after CNO injection at day 14 in these rats (Fig. 5E). It is worth noting that the absolute value of the ΔHWT in the hM3Dq-expressing PEOI group was significantly greater than that in the hM3Dq-expressing REOI group, indicating that a greater reduction in the HWTs occurred in these rats (Fig. 5E). Only the rats with the injection sites within the RVM were included in the analysis (Fig. 5F). The infection rate and specificity of these viruses in the RVM were verified by immunofluorescence staining (Fig. 5F). The infection rate of these viruses was approximately 82.1% and the specificity of the Tph2 promoter was approximately 73.8% (Fig. 5G, H).

In summary, chemogenetic inhibition of RVM 5-HT neurons could only reverse the reduced HWTs on day 14 of REOI rats, whereas chemogenetic activation of RVM 5-HT neurons could further decrease the reduced HWTs on day 14 of both REOI and PEOI rats.

### Selective down-regulation of 5-HT synthesized in the RVM could reverse hyperalgesia after delayed EOI removal but did not alter hyperalgesia associated with persistent EOI

**Fig. 6:**
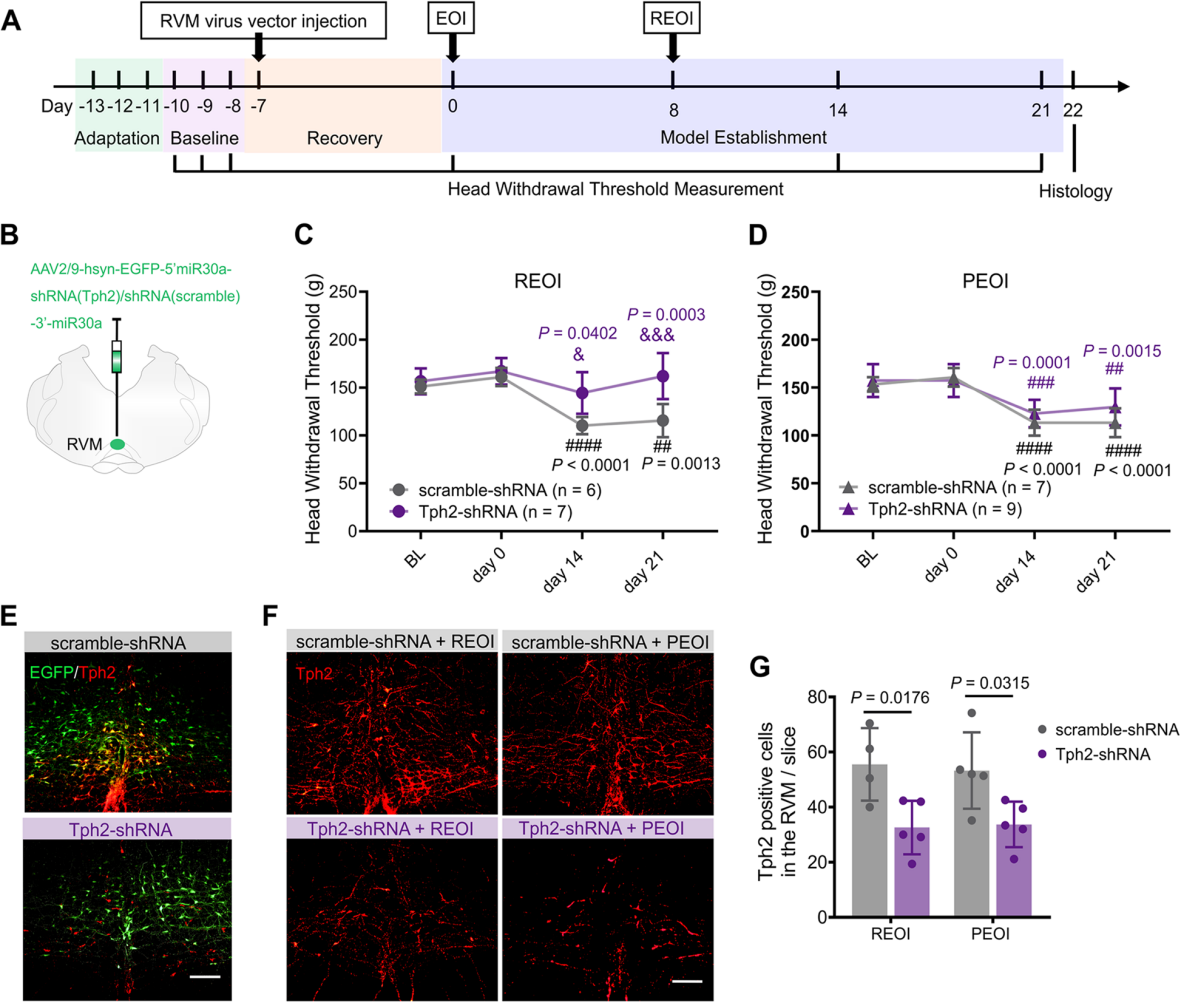
Downregulation of 5-HT synthesis reversed hyperalgesia after delayed REOI but did not alter PEOI-induced hyperalgesia. **A** Schematic diagram of the experimental time course of HWT measurements (*n* = 7–9 in each group). **B** Injection of viruses into the RVM region. **C**, **D** Changes in HWTs in all the groups. Results represent mean ± SD; two-way repeated measures ANOVA, followed by Tukey’s multiple-comparison test. & *P* < 0.05, &&& *P* < 0.001, Tph2-shRNA-expressing REOI group vs. scramble-shRNA-expressing REOI group. ## *P* < 0.01, ### *P* < 0.001, #### *P* < 0.0001, day 14 or day 21 vs. baseline. **E** Representative images revealed co-expression of Tph2-labeled neurons with EGFP in the RVM in the scramble-shRNA-expressing rats while little co-expression was observed in the Tph2-shRNA-expressing rats. Scale bar, 200 μm. **F** Immunofluorescence staining illustrated the number of Tph2-positive neurons in the Tph2-shRNA-expressing and the scramble-expressing REOI groups, as well as in the Tph2-shRNA-expressing and the scramble-shRNA-expressing PEOI groups. Scale bar, 200 μm. **G** Semi-quantitative analysis of the number of Tph2-positive cells in all the groups. Fewer Tph2-positive cells were found in Tph2-shRNA-expressing REOI group (vs. scramble-shRNA-expressing REOI group) and Tph2-shRNA-expressing PEOI group (vs. scramble-shRNA-expressing PEOI group). Results represent mean ± SD; two-way ANOVA, followed by Tukey’s multiple-comparison test. Tph2, tryptophan hydroxylase 2

To evaluate the role of 5-HT synthesized in the RVM on the hyperalgesia after delayed EOI removal or after persistent EOI, we also interfered with the synthesis of 5-HT by performing the RNA interference of Tph2. Behavioral tests were conducted in REOI and PEOI groups that received an injection of Tph2-shRNA or scramble virus into the RVM before EOI placement (Fig. 6A, B).

The HWTs were not changed after the virus injection surgery in all groups (Fig. 6C, D). The main effect of time (*F*_*(1.883, 20.71)*_ = 20.86 *P* < 0.0001), virus type (*F*_*(1, 11)*_ = 12.89, *P* = 0.0042), and their interaction (*F*_*(3, 33)*_ = 8.412, *P* < 0.0003) on the HWT of REOI rats were significant. The main effects of time (*F*_*(1.784, 24.97)*_ = 47.38, *P* < 0.0001) and the interaction of time and virus type (*F*_*(3, 33)*_ = 8.412, *P* < 0.0003) on the HWT of PEOI rats were significant, while the main effect of virus type (*F*_*(1, 14)*_ = 1.603, *P* = 0.2262) was not significant. The HWTs were significantly reduced on days 14 and 21 in the scramble-shRNA-expressing REOI group (Fig. 6C) and the scramble-shRNA-expressing PEOI group (Fig. 6D). However, the Tph2-shRNA-expressing REOI group did not show significantly reduced HWTs on days 14 and 21 (Fig. 6C). On the contrary, the HWTs were still significantly reduced in the Tph2-shRNA-expressing PEOI group on days 14 and 21 (Fig. 6D).

We also analyzed the effect of scramble-shRNA or Tph2-shRNA on the expression of Tph2 in the RVM of REOI and PEOI rats, respectively. The co-expression of EGFP and Tph2 in the RVM of scramble-shRNA-expressing rats was verified histologically, whereas few EGFP was co-expressed with Tph2 in the RVM of Tph2-shRNA-expressing rats (Fig. 6E). A significant decrease in Tph2 expression was observed in the Tph2-shRNA-expressing REOI and the Tph2-shRNA-expressing PEOI groups (Fig. 6F). The factor of virus type (*F*_*(1, 15)*_ = 16.43, *p* = 0.0010) showed a significant main effect on the number of Tph2-positive cells in the RVM. Further analysis revealed that the number of Tph2-positive cells in the RVM was significantly decreased in the Tph2-shRNA-expressing REOI group compared to the scramble-shRNA-expressing REOI group (Fig. 6G). Similar findings were revealed in the Tph2-shRNA-expressing PEOI group; the number of Tph2-positive cells in the RVM of these rats was also significantly decreased compared to the scramble-shRNA-expressing PEOI group (Fig. 6G). These results indicate that Tph2-shRNA virus injection effectively downregulated the synthesis of 5-HT in the RVM of both REOI and PEOI rats.

## Discussion

Consistent with our previous findings [25, 26], this study documented that both persistent EOI and delayed removal of EOI are associated with the maintenance of chronic myofascial orofacial hyperalgesia. The mechanisms for the development of chronic myofascial orofacial hyperalgesia in these models involve central sensitization in the Sp5C [7] and unbalanced descending modulation in the RVM [27, 28]. The development of hyperalgesia after nerve injury or inflammation has been shown to involve central mechanisms in the Sp5C induced by peripheral nociceptive afferent inputs [8, 31, 36–38]. It is also noteworthy that a delayed descending 5-HT-driven central sensitization in the Sp5, together with persistent peripheral inputs, has been shown to underlie the maintenance phase of secondary hyperalgesia induced by nerve injury [31]. Nevertheless, it is difficult to precisely distinguish these descending influences on the maintenance of hyperalgesia with or without persistent peripheral input.

The REOI model in our study was able to separate the effect of the peripheral stimulus from the descending modulatory drive on the central mechanisms by removing the EOI at a stage when the hyperalgesia becomes chronic [27]. The study first demonstrated that the 5-HT pathway from the RVM to the Sp5 facilitates the chronic myofascial orofacial hyperalgesia after delayed EOI removal, but plays a minor role in persistent EOI-induced chronic myofascial orofacial hyperalgesia.

Earlier studies have provided evidence that RVM stimulation modulates the activity of Sp5C nociceptive neurons and orofacial nociceptive behavior [39–41] and that the descending 5-HT pathway modulates nociceptive activity in Sp5C [31, 42]. In particular, some early studies showing the involvement of 5-HT3 receptor in the facilitation of pain behavior mainly focused on the behavioral effects of 5-HT3 receptor ligands (mostly antagonists) [23, 29, 30, 36]. In this study, the expression levels of both 5-HT3A and 5-HT3B receptor subtypes in the Sp5 were measured. Both persistent EOI and delayed EOI removal were found to upregulate the 5-HT3B receptor subtype, but did not change the expression level of the 5-HT3A receptor subtype. Interestingly, the 5-HT3B receptor subtype may preferentially form the heteromeric 5-HT3AB receptor [43]. However, there are no 5-HT3B knock-out mice or selective 5-HT3B antagonists that are able to differentiate the homomeric 5-HT3A from the heteromeric 5-HT3AB receptors yet. Therefore, further research on elucidating the structural and functional characteristics of the heteromeric 5-HT3AB receptor is needed.

We further found that antagonizing 5-HT3 receptors in the Sp5 dose-dependently reversed the hyperalgesia after delayed EOI removal, indicating that the upregulation of 5-HT3 receptors in the Sp5 may correlate with net descending facilitation. This result is also similar to previous findings that spinal or trigeminal 5-HT3 receptors mediate pain facilitation [15, 20, 30]. We also found that RVM 5-HT neurons exert a pronociceptive influence in the maintenance of hyperalgesia after delayed EOI removal, consistent with the descending facilitatory effect produced by RVM 5-HT neurons in other pain models [15, 17, 31, 44]. Taken together, these findings indicate that the descending 5-HT output from the RVM may act on the 5-HT3 receptors in the Sp5 and thus maintain trigeminal central sensitization reflected in neuronal hyperexcitability, glial hyperactivity and so on [15, 31].

Interestingly, the present results also suggest that the RVM descending 5-HT pathway does not exert a major pronociceptive influence in maintaining the myofascial orofacial hyperalgesia after persistent EOI, since neither chemogenetic inhibition of RVM 5-HT neurons nor downregulation of 5-HT synthesis in the RVM relieved the hyperalgesia after persistent EOI. Although the expression level of the 5-HT3B receptor subtype in the Sp5 increased after persistent EOI, selective antagonism of the 5-HT3 receptor only transiently relieved the hyperalgesia, suggesting a minor role of the 5-HT3 receptor in maintaining the hyperalgesia after persistent EOI. We showed that activation of the RVM 5-HT neurons led to an exacerbation of hyperalgesia after persistent EOI, whereas chemogenetic inhibition of the RVM 5-HT neurons did not affect the hyperalgesia, suggesting that RVM 5-HT neurons may not be activated after persistent EOI. This result is similar to the finding that RVM descending pain inhibition rather than facilitation predominates in the maintenance of the hyperalgesia after persistent EOI [27]. The chronic myofascial orofacial hyperalgesia after persistent EOI may be modulated by higher-level brain regions that are also involved in descending modulation [45–47].

There are several limitations to this study. Firstly, the specific neuronal subpopulation or nerve fiber type within the Sp5 that received the descending 5-HT modulation was not determined, such as the Sp5 pain-transmission neurons, inhibitory interneurons, excitatory interneurons, or terminals of primary sensory neurons [13, 15]. Secondly, the present study was conducted only on male rats, so whether the present findings could also apply to females needs to be determined in future studies. Future research is also needed to elucidate the role of other 5-HT receptor subtypes, especially the analgesic receptor subtypes (such as the 5-HT2A or 5-HT2C receptor subtypes), in EOI-associated chronic myofascial orofacial hyperalgesia. As the present study and previous studies [27, 28] revealed a limited descending modulatory role of the RVM in persistent EOI-associated hyperalgesia, the role of higher-level brain regions in modulating chronic myofascial orofacial pain also needs further investigation.

## Conclusions

In conclusion, we have demonstrated that the descending neurotransmitter from 5-HT neurons in the RVM to 5-HT3 receptors in the Sp5, exerts a facilitatory descending modulatory influence on the maintenance of myofascial orofacial hyperalgesia after delayed removal of EOI, but plays a limited role in the hyperalgesia after persistent EOI. The present results also have provided evidence that the maintenance of myofascial orofacial pain with or without persistent peripheral stimulus involves different descending modulatory mechanisms. Further studies are needed to investigate the potential differences in descending modulatory mechanisms in these two situations.

## Supplementary Information

**Additional file 1: Figure S1.** Rat acclimatization and head withdrawal threshold measurement. **Table S1.** Summary of statistical analyses. **Figure S2.** Full unedited gels for Figs. 1F and 2F. **Figure S3.** GABAergic and glutaminergic neurons receiving inputs from the RVM colocalize with 5-HT3B receptor subtype in the Sp5 respectively.

## Data Availability

The data supporting the findings of this study are available within the article and its supplementary material. Inquiries for additional data are available from the corresponding authors upon reasonable request.

## Abbreviations

Sp5: Spinal trigeminal nucleus
RVM: Rostral ventromedial medulla
5-HT: Serotonin
EOI: Experimental occlusal interference
PEOI: Persistent EOI
REOI: Removal of EOI
HWT: Head withdrawal threshold
PBS: Phosphate-buffered saline
HPLC: High-performance liquid chromatography
5-HIAA: 5-Hydroxy indole acetic acid
aCSF: Artificial cerebrospinal fluid
CNO: Clozapine oxide
Tph2: Tryptophan hydroxylase 2
Sp5C: Subnucleus caudalis
Sp5I/Sp5C: Subnucleus interpolaris/subnucleus caudalis transition zone
Sp5I: Subnucleus interpolaris
