# Supplementary material for: Descending serotonergic modulation from rostral ventromedial medulla to spinal trigeminal nucleus is involved in experimental occlusal interference-induced chronic orofacial hyperalgesia
Source: J Headache Pain. 2023 May 10;24(1):50. doi: 10.1186/s10194-023-01584-3 (PMC10173589; doi:10.1186/s10194-023-01584-3)
Supplement: Supplementary file 1 — Additional file 1: Figure S1. Rat acclimatization and head withdrawal threshold measurement. Table S1. Summary of statistical analyses. Figure S2. Full unedited gels for Figs. 1F and 2F. Figure S3. GABAergic and glutaminergic neurons receiving inputs from the RVM colocalize with 5-HT3B receptor subtype in the Sp5 respectively. [file 10194_2023_1584_MOESM1_ESM.docx]

**Descending serotonergic modulation from rostral ventromedial medulla to spinal trigeminal nucleus ~~is~~ involved in experimental occlusal interference-induced chronic orofacial hyperalgesia**


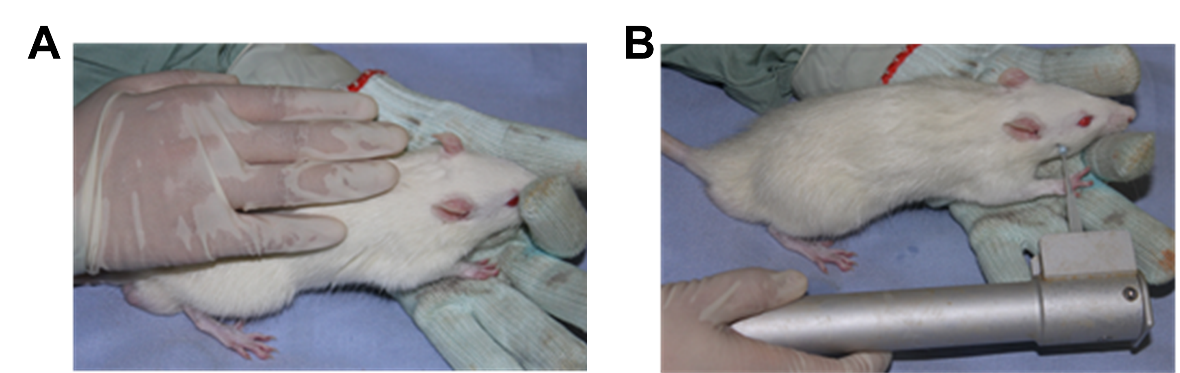
Si-Yi Mo^1,2^, Yang Xue^1,2^, Yuan Li^1,2^, Yao-Jun Zhang^1,2^, Xiao-Xiang Xu^1,2,*^, Kai-Yuan Fu^2,4^, Barry J. Sessle^5^, Qiu-Fei Xie^1,2^, and Ye Cao^1,2,3,*^

Figure S1 Rat acclimatization (A) and head withdrawal threshold measurement (B).

Table S1 Summary of statistical analyses

| Figure | Analysis | Interaction P | Interaction F | N/group |
| --- | --- | --- | --- | --- |
| 1C | One-way ANOVA-Tukey | P = 0.6477 | F (2, 6) = 0.4673 | 3 |
| 1D | One-way ANOVA-Tukey | P = 0.8165 | F (2, 6) = 0.2097 | 3 |
| 1E | One-way ANOVA-Tukey | P = 0.9905 | F (2, 6) = 0.009532 | 3 |
| 1F | One-way ANOVA-Tukey | P = 0.5824 | F (2, 12) = 0.5657 | 5 |
| 2C | One-way ANOVA-Tukey | P = 0.0216 | F (2, 6) = 7.768 | 3 |
| 2D | One-way ANOVA-Tukey | P = 0.9905 | F (2, 6) = 0.009532 | 3 |
| 2E | One-way ANOVA-Tukey | P = 0.0050 | F (2, 6) = 14.56 | 3 |
| 2F | One-way ANOVA-Tukey | P = 0.0031 | F (2, 12) = 9.754 | 5 |
| 3C | One-way ANOVA-Tukey | P = 0.0053 | F (2, 9) = 9.923 | 4 |
| 3D | One-way ANOVA-Tukey | P = 0.0125 | F (2, 9) = 7.418 | 4 |
| 3E | One-way ANOVA-Tukey | P = 0.0020 | F (2, 9) = 13.44 | 4 |
| 3F | One-way ANOVA-Tukey | P = 0.0249 | F (2, 9) = 5.721 | 4 |
| 4B | RM Three-way ANOVA - Tukey | P < 0.0001 | F (15, 114) = 1.457 | 6-8 |
| 4C | RM Three-way ANOVA - Tukey | P < 0.001 | F (9.006, 58) = 10.580 | 6-7 |
| 5C | RM Two-way ANOVA - Tukey | P < 0.0001 | F (10, 90) = 17.25 | 6-8 |
| 5D | RM Two-way ANOVA - Tukey | P < 0.0001 | F (10, 90) = 9.708 | 6-8 |
| 5E | Two-way ANOVA - Tukey | P < 0.0001 | F (2, 36) = 14.97 | 6-8 |
| 6C | RM Two-way ANOVA - Tukey | P = 0.0003 | F (3, 33) = 8.412 | 6-7 |
| 6D | RM Two-way ANOVA - Tukey | P = 0.1643 | F (3, 42) = 1.787 | 7-9 |
| 6G | Two-way ANOVA - Tukey | P = 0.7499 | F (1, 15) = 0.1054 | 4-5 |


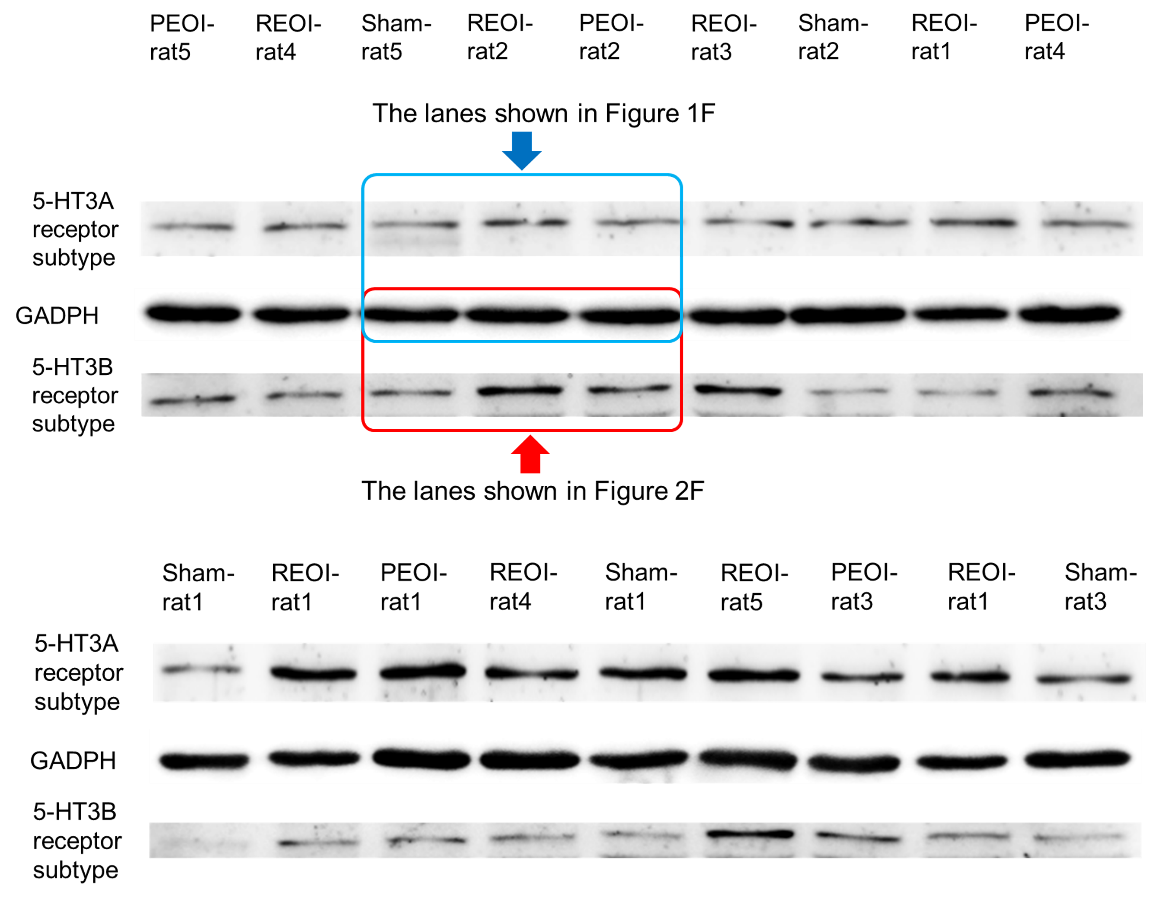


Figure S2 Full unedited gels for Figure 1F and Figure 2F.


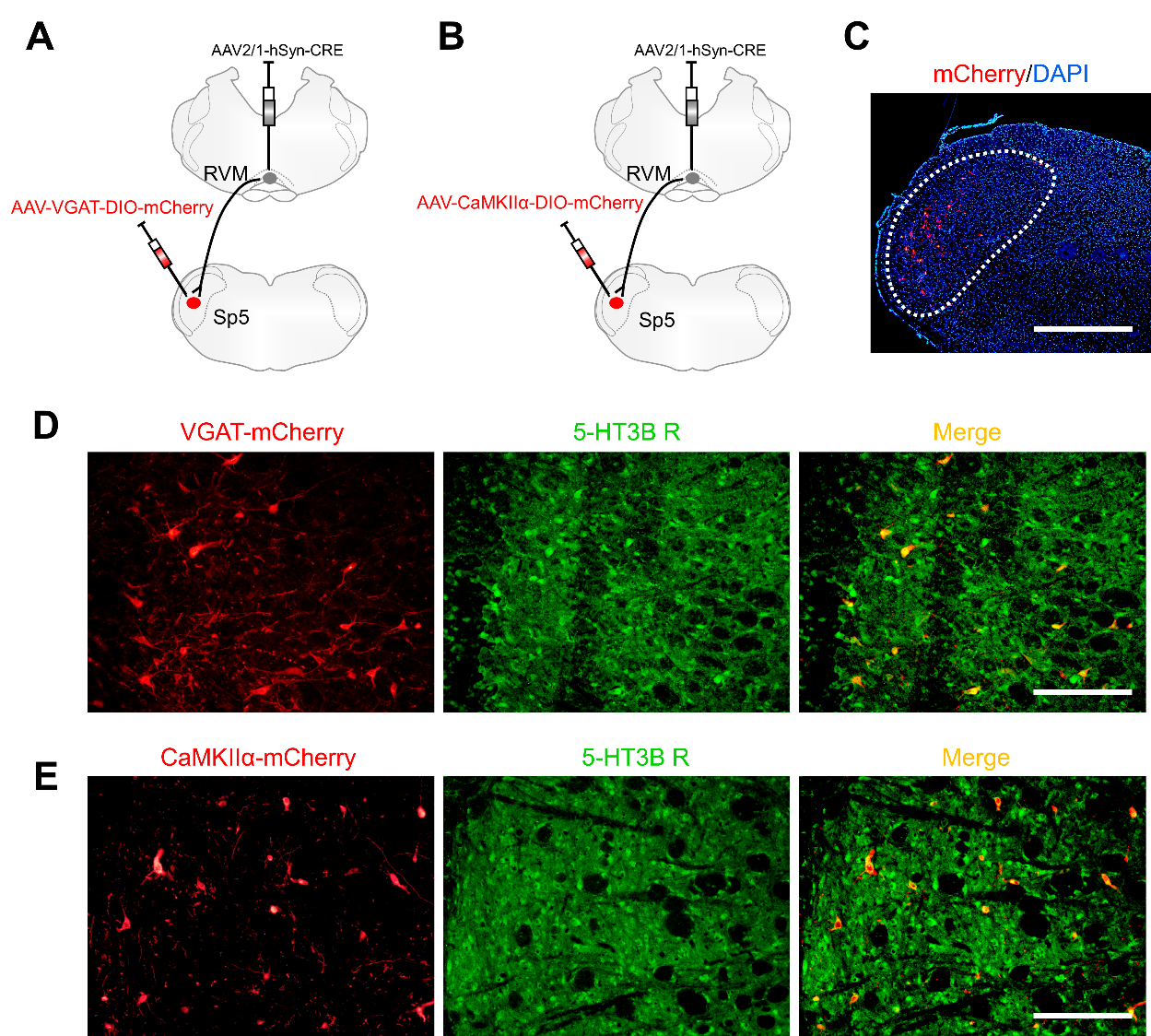


Figure S3 GABAergic and glutaminergic neurons receiving inputs from the RVM colocalize with 5-HT3B receptor subtype in the Sp5 respectively. (A, B) Injection of AAV2/1-hSyn-CRE virus in the RVM, AAV-VGAT-DIO-mCherry or AAV-CaMKIIα-DIO-mCherry virus in the right Sp5. (C) Distribution of glutaminergic neurons receiving inputs from the RVM in the Sp5. Scale bar, 1000 μm. (D) GABAergic neurons receiving inputs from the RVM colocalize with 5-HT3B receptor subtype. Scale bar, 200 μm. (D) Glutaminergic neurons receiving inputs from the RVM colocalize with 5-HT3B receptor subtype. Scale bar, 200 μm.
